# Unveiling the genetic biomarkers for ageing: evidence from a large sample genome-wide association study and in vivo validation

**DOI:** 10.7189/jogh.15.04279

**Published:** 2025-09-26

**Authors:** Zhikang Cai, Yue Yang, Peng Qu, Sensong Fu, Xu Li

**Affiliations:** 1 Department of Emergency Medicine, Nanfang Hospital, Southern Medical University, Guangzhou, China; 2 Department of Obstetrics and Gynecology, Nanfang Hospital, Southern Medical University, Guangzhou, China; 3 Department of Gastroenterology, Nanfang Hospital, Southern Medical University, Guangzhou, China; 4 Guangdong Provincial Key Laboratory of Gastroenterology, Department of Gastroenterology, Nanfang Hospital, Southern Medical University, Guangzhou, China; 5 Department of Neuro-oncological Surgery, Zhujiang Hospital, Southern Medical University, Guangzhou, China; 6 Department of Clinical Research Center, Nanfang Hospital, Southern Medical University, Guangzhou, China

## Abstract

**Background:**

Ageing, marked by cumulative molecular damage, now leaves most adults spending nearly a decade in poor health. To date, no therapies directly target the ageing process. We performed a large-scale genome-wide association study to identify potential drug targets for extending health span.

**Methods:**

By combining genetic and experimental evidence, we prioritise therapeutic targets with the potential to extend healthy lifespan. Using two-sample Mendelian randomisation (MR) across 26 152 expression quantitative trait loci instruments, we screened for causal links between 5430 potential drug target genes and four ageing phenotypes – frailty index (n = 175 226), HannumAge (n = 34 710), intrinsic epigenetic age acceleration (n = 34 710), and telomere length (n = 742 174). We re-evaluated high-confidence loci with summary-databased MR (SMR) and validated them by quantitative polymerase chain reaction (qPCR), Nissl staining, and Western blotting in three- and 20-month-old C57BL/6 mice. Finally, replication in a meta genome-wide association study (GWAS) of long-lived individuals *vs.* controls across 20 diverse cohorts upheld the association. This integrated genetic-experimental strategy prioritises candidate therapeutic targets for interventions aimed at extending healthy lifespan.

**Results:**

Two-sample MR mapped 47 gene-ageing links spanning frailty, telomere length, and two epigenetic clocks. The SMR confirmed 11 with consistent directions and heterogeneity in the dependent instrument support. Both qPCR and Western blot in three- and 20-month C57BL/6 mice confirmed age-dependent down-regulation of UBA7, PLA2G4B, and ATP8B4, validating their functional relevance. Finally, replication in a longevity meta-GWAS specifically confirmed the association for UBA7.

**Conclusions:**

These findings highlight UBA7, PLA2G4B, and ATP8B4 as promising targets for interventions aimed at extending health span, shedding light on the biological mechanisms of longevity.

Ageing is a biological process characterised by the progressive accumulation of cellular and molecular damage, resulting in declining physiological function, increased disease susceptibility, and mortality [1,2]. Advances in nutrition, hygiene, immunisation, antibiotics, and healthcare over the past century have contributed to a substantial increase in life expectancy in developed nations [3,4]. As a result, the global population is undergoing a significant demographic shift toward an ageing society. In 2020, there were approximately 962 million individuals aged ≥60 years, and this number is projected to approach two billion by 2050. Notably, older adults will, for the first time in history, surpass the number of adolescents and young adults globally [5,6]. This demographic shift marks a significant milestone in longevity but also poses substantial challenges to societal, economic, and healthcare systems, as increased life expectancy does not necessarily ensure prolonged periods of good health. These challenges are especially pronounced in developing countries [7]. Contrary to the assumption that longevity improvements reflect parallel gains in overall health, recent evidence suggests limited improvements in morbidity profiles among contemporary older adults compared to previous generations [8]. Over 90% of individuals aged ≥65 years have at least one chronic condition, and more than 70% contend with multiple chronic diseases, such as arthritis, diabetes, cancer, heart disease, and stroke [9,10]. Moreover, ageing itself constitutes a major risk factor for the onset of chronic diseases [11]. Consequently, interventions targeting the underlying biological processes of ageing may prove more effective in improving health outcomes for older adults than strategies aimed solely at individual diseases. However, progress in developing anti-ageing therapeutics has been slow, as thousands of candidate compounds have failed to demonstrate efficacy in modulating ageing-related mechanisms [12]. This highlights the urgent need to prioritise the identification and validation of novel therapeutic targets that can enhance health span and alleviate the burden of age-related diseases [13].

Drug development, although transformative in medical practice and pivotal in reducing disease burden over the past century [14], continues to face significant challenges, including prohibitive costs and high failure rates – fewer than 10% of drug candidates progress successfully through clinical trials [15,16]. A critical factor contributing to this high attrition rate is the inadequate prediction of efficacy during early-stage target selection [17]. To address this challenge, a growing strategy in drug target investigation leverages genome-wide association studies (GWASs), as many biologics are designed to target proteins encoded by specific genes [18,19]. Evidence indicates that therapeutics designed against genetically validated targets have a higher likelihood of clinical success, with success rates potentially doubling from phase I to market approval [17,19]. For example, beyond the successful application of PCSK9 inhibitors, cholesteryl ester transfer protein inhibitors identified through such genetic approaches are currently undergoing phase III clinical trials [20], demonstrating the remarkable potential of human genetic data to drive therapeutic advancements.

To comprehensively explore ageing-related drug targets, we employed expression quantitative trait loci (eQTLs) as exposure variables and selected three key biomarkers – frailty index (FI), first-generation epigenetic clocks (HannumAge and intrinsic epigenetic age acceleration (IEAA)) [20], and telomere length (TL) – because these indicators more accurately represent biological age (BA). Unlike chronological age (CA), which is solely based on birthdate and does not account for genetic, environmental, and lifestyle factors that significantly influence ageing, BA integrates multiple biological dimensions. This integration enhances the identification of individuals with accelerated ageing and facilitates the discovery of potential drug targets for ageing-related diseases. First, the FI, a macro-level indicator assessing accumulated health deficits, offers superior predictive power over CA for outcomes associated with physiological ageing [20,21]. The DNA methylation age, often referred to as the ‘epigenetic clock’, is derived from DNA methylation profiles at specific cytosine-phosphate-guanine sites (*i.e.* 5′-C-phosphate-G-3′) and is widely recognised as a promising biomarker of biological ageing [22]. We used two widely validated first-generation epigenetic clocks – HannumAge [23] and IEAA [24] – which were constructed from DNA levels at cytosine-phosphate-guanine loci identified as being highly correlated with BA, enabling precise estimation of CA. Finally, TL is a reliable proxy for BA because it progressively shortens with each cell division and accumulates damage from oxidative stress, directly reflecting cellular replicative history and genomic integrity over time [25]. Its shortening is closely linked to an elevated risk of numerous age-related diseases [26]. By integrating these multi-level indicators, we aim to elucidate the complex mechanisms underlying physiological ageing, identify potential drug targets, and develop novel strategies to delay ageing and prevent age-related diseases.

## METHODS

We adhered to the Journal of Global Health’s Guidelines for Reporting Analyses of Big Data Repositories Open to the Public (Table S1 in the **Online Supplementary Document**) [27].

### Study framework

**Figure 1:**
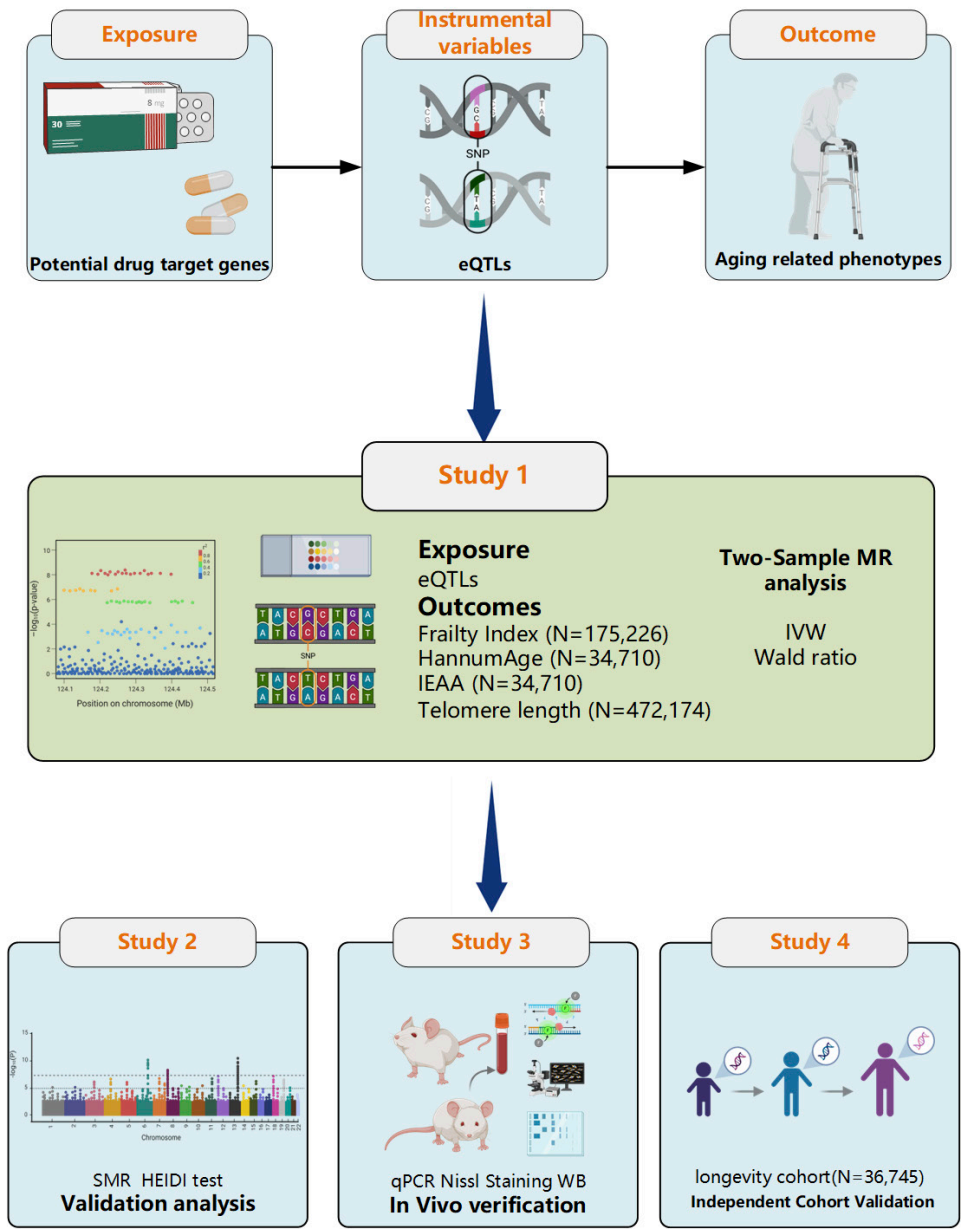
Study framework.

To identify robust therapeutic targets for mitigating ageing-related processes, we employed a multi-stage analytical framework (**Figure 1**). First, we performed a two-sample Mendelian randomisation (MR) analysis to assess the causal effects of genetic variants on ageing phenotypes, including FI, HannumAge, IEAA, and TL. Subsequently, we conducted a summary-databased MR (SMR) analysis to prioritise candidate targets exhibiting strong associations. We validated candidate targets that showed consistent signals at the mRNA level using quantitative polymerase chain reaction (qPCR), and further confirmed those with concordant expression trends through Western blot. In addition, we used an independent cohort to verify the reproducibility and generalisability of the findings. Finally, we performed phenome-wide association analysis to reveal diseases correlated with identified ageing genes.

### MR

#### Data source for exposure and instrument variable selection

We derived eQTLs from RNA sequencing data from 31 684 individuals [28]. We selected genetic variants that exhibited strong associations with the exposure, meeting the genome-wide significance threshold (*P* < 5 × 10^−8^), as instrumental variables (IVs) to ensure robust relevance for subsequent analyses [29]. To confirm the independence of these variants and reduce potential confounding due to linkage disequilibrium, we applied strict clumping parameters (r^2 ^< 0.001) within a 1 megabase genomic window [30]. Additionally, to minimise weak instrument bias, we retained only single-nucleotide polymorphisms (SNPs) with an F-statistic >10, thus ensuring the validity and power of the selected IVs for downstream MR analyses (Table S2 in the **Online Supplementary Document**) [31].

#### Data source for ageing-related outcomes

Genetic data for FI were retrieved from the Integrative Epidemiology Unit (IEU) GWAS database, which included 175 226 individuals of European ancestry [32]. Genetic instruments for TL were derived from GWAS data involving 472 174 individuals of European origin, sourced from publicly available data sets curated by the IEU [33]. Additionally, data on IEAA, HannumAge were obtained from a large-scale GWAS meta-analysis involving 34 710 individuals of European ancestry (Table S3 in the **Online Supplementary Document**) [34].

#### MR analysis and sensitivity analysis

Genetic variants can be used as IVs in MR to reduce confounding and reverse causation. This approach relies on three key assumptions: relevance – the IVs are strongly associated with the exposure; independence – the IVs are not associated with confounders; and exclusion restriction – the IVs influence the outcome solely through the exposure. We used four complementary MR methods to assess causal effects. For genes with a single SNP, we applied the Wald ratio method to estimate the causal effect by calculating the ratio of the SNP’s effect on the outcome to its effect on the exposure. For genes with ≥2 SNPs, we used the inverse variance weighted method to estimate the causal effect [35]. To enhance robustness and address potential pleiotropy, we supplemented the inverse variance weighted estimates with MR-Egger regression and weighted median [36]. To evaluate potential horizontal pleiotropy, we performed an MR-Egger regression and assessed the intercept. A non-significant intercept (*P* > 0.05) indicates the absence of directional pleiotropy, suggesting that pleiotropic effects do not influence the IVs.

#### SMR and heterogeneity in the dependent instrument (HEIDI) test

To address potential horizontal pleiotropy, we conducted an SMR analysis (*P* < 0.05), which supported our core MR results. Additionally, we performed the HEIDI test alongside the SMR analysis using the SMR software tool [37], which showed a uniform genetic effect (*P* > 0.05) and excluded linkage disequilibrium.

#### Animals

We obtained male C57BL/6 mice at ages of three and 20 months from the SPF (Beijing) Biotechnology Co., Ltd We housed the animals under specific pathogen-free conditions with free access to standard chow and water. The Institutional Animal Care and Use Committee of Southern Medical University approved all animal experiments (reference number: SMUL202504042).

#### qPCR

We collected peripheral blood from three-month-old (n = 6) and 20-month-old mice (n = 6) via the tail vein using heparinised capillaries. We isolated total RNA from blood using the Whole Blood RNA Extraction Kit (Sangon Biotech, #B518653). We reverse transcribed RNA into cDNA using the PrimeScript RT Kit (Sangon Biotech, #B639252). We performed reverse transcription-qPCR with SYBR Green PCR Master Mix (Sangon Biotech, #B690016) and gene-specific primers. We calculated relative gene expression using the 2^−ΔΔCt^ method (Table S4 in the **Online Supplementary Document**).

#### Nissl staining

We fixed brain tissues (n/N = 3/group) in 4% paraformaldehyde for 48 hours, dehydrated, embedded, and sectioned them at 4 μm thickness. Sections underwent deparaffinisation, rehydration, and Nissl staining (Shanghai Wknow Bio-tech, WK00077) for 5 minutes, followed by rapid dehydration and drying. After clearing with xylene for 10 minutes, we mounted the sections using neutral resin. We examined tissue morphology under a high-power microscope and quantified Nissl body areas in ImageJ software.

#### Western blotting

We lysed the tissues (n/N = 10/group) in RIPA Lysis Buffer (Beyotime, P0013B) for total protein extraction, and quantified protein concentrations using the BCA Protein Assay Kit (Beyotime, P0011). We denatured protein samples at 100°C for 15 minutes. We loaded equal amounts of protein onto SDS-PAGE gels (Epizyme, PG122) and electrophoresed them at 90 V for 30 minutes, followed by 120 V for 60 minutes. We then transferred the proteins onto polyvinylidene fluoride membranes (Millipore, IPVH00010) at a constant current of 280 mA for 90 minutes. Subsequently, we blocked the membranes using 5% skim milk and incubated them with primary antibodies targeting UBA7 (Abmart, T59622), PLA2G4B (Affinity, DF9429), and GAPDH (Proteintech, 10494-1-AP) overnight at 4°C. After washing three times with Tris-buffered saline with Tween-20, we incubated the membranes with horseradish peroxidase-conjugated secondary antibodies at room temperature for 1 hour. We acquired the images using a chemiluminescence imaging system and quantified them using ImageJ software.

#### Independent cohort validation

To verify the reliability of our results, we conducted a replication analysis using GWAS data from longevity cohorts, defining the phenotype as survival beyond the 90th percentile. Specifically, we collected meta-analyses of GWAS data from 20 cohorts representing European, East Asian, and African American populations, with cases defined as individuals exceeding the 90th survival percentiles (n = 11 262) – criteria derived from standardised life table analyses – while 25 483 participants below the 60th percentile were designated as controls [38]. Subsequently, we applied two-sample MR and compared the results with our previous findings, thereby further substantiating the reliability of our conclusions. Furthermore, we performed a phenome-wide MR analysis, using the identified candidate drug-target genes as exposures and systematically evaluating their causal effects on 237 age-related disease phenotypes [39] derived from FinnGen R12 and the IEU Open GWAS catalogue. These phenotypes were grouped into 10 systems (Table S5 in the **Online Supplementary Document**). We conducted a two-sample MR analysis for causal inference, with associations of *P* < 0.05 considered statistically significant.

### Statistics analysis

To ensure rigorous control of Type I error across extensive multiple testing, we employed Bonferroni corrections to adjust the significance thresholds. For the analysis, we calculated the adjusted thresholds as: *P* < 0.05 / (5425 × 4) = 2.3 × 10^−6^ for FI, *P* < 0.05 / (5429 × 4) = 2.3 × 10^−6^ for TL, *P* < 0.05 / (5428 × 4) = 2.3 × 10^−6^ for IEAA, *P* < 0.05 / (5428 × 4) = 2.3 × 10^−6^ for Hannum. We applied the thresholds to ensure robustness and enhance the study’s credibility and reproducibility. We used the 'TwoSampleMR' package, version 0.5.11 in *R*, version 4.3.3 (R Core Team, Vienna, Austria) for all analyses. For in vivo verification, we used an unpaired *t*-test with Welch's correction between two groups. We presented the results as means (standard error of the mean), and considered *P* < 0.05 as statistical significance.

## RESULTS

### Biomarkers for ageing: evidence from large sample GWAS analysis

We found seven genes associated with FI using two-sample MR, among which four were protective genes for ageing and three were risk genes (**Table 1**). For FI, UBA7, TNF, APEH, and LRPPRC were protective factors, and TNXB, CRLF3, and SUZ12P1 were risk factors for ageing. For TL, CTC1, LINC00324, APEH, CLEC18A, STAG3, VARS2, WDR81, PLA2G4B, ATP8B4, MPHOSPH6, COG4, HCG11, UBA7 were protective factors, and TGS1, KMT5A, HCG9, POLI, HEATR3, STN1, IQCG, and RPA2 were risk factor for ageing. For IEAA, ATP8B4 and H2BC18 were protective factors for ageing. For Hannum, LINC00243 was a risk factor, while CD248 and HLA-L were protective factors for ageing. Sensitivity analyses reinforced the robustness of these phenotype causal associations (Figure S1 in the **Online Supplementary Document**). Likewise, the MR-PRESSO global test detected no influential outliers or pleiotropy (Table S6 in the **Online Supplementary Document**).

**Table 1:** Significant two-sample MR findings between genes and the four ageing-related traits

	Gene	SNPs	OR (95% CI)	*P*-value
**FI**	TNXB	rs2844503, rs2269426	1.070 (1.046–1.094)	2.36^−09^
	UBA7	rs9469079, rs116375541, rs76298543	0.965 (0.954–0.977)	4.87^−09^
	TNF	rs13086611, rs2236939	0.938 (0.918–0.959)	1.35^−08^
	APEH	rs1121800, rs72855945	0.953 (0.936–0.971)	1.63^−07^
	CRLF3	rs9469017, rs115437298, rs76298543, rs13064576	1.042 (1.025–1.058)	5.81^−07^
	SUZ12P1	rs149007767, rs216412	1.048 (1.028–1.068)	8.76^−07^
	LRPPRC	rs9900596, rs7405606, rs34086083, rs34086083, rs7213433, rs1061342, rs4073237, rs6720846, rs115672688	0.964 (0.950–0.979)	1.14^−06^
**IEAA**	ATP8B4	rs12485444, rs149007767, rs7846314, rs2497306, rs35609972, rs75217875, rs937171	0.723 (0.647–0.807)	9.64^−09^
	H2BC18	rs2413974, rs192504603, rs2664717, rs11212617	0.342 (0.221–0.528)	1.42^−06^
**Hannum**	CD248	rs6457374, rs678347	0.429 (0.313–0.588)	1.50^−07^
	LINC00243	rs565972, rs3131781, rs1131114	1.461 (1.253–1.703)	1.28^−06^
	HLA-L	rs145227731, rs758778, rs9468618, rs2523609, rs7383281, rs9263993, rs9261293	0.719 (0.627–0.824)	2.23^−06^
**TL**	TGS1	rs56224379, rs7836019	0.962 (0.954–0.970)	5.57^−20^
	COG4	rs10101332, rs2549242	1.054 (1.041–1.067)	3.38^−17^
	KMT5A	rs9746247, rs7192865	0.956 (0.946–0.966)	1.41^−16^
	WDR81	rs7139321, rs6488882	1.037 (1.026–1.048)	5.30^−12^
	VARS2	rs28780730, rs3976, rs59096313, rs59282480	1.033 (1.024–1.043)	1.05^−11^
	CLEC18A	rs1345230, rs1264345	1.029 (1.020–1.038)	2.10^−10^
	HCG9	rs114475062, rs111511435, rs73425709	0.960 (0.947–0.972)	2.13^−10^
	MPHOSPH6	rs78203089, rs1006985, rs4985376, rs9258357	1.053 (1.035–1.070)	1.44^−09^
	POLI	rs9468618, rs2735071, rs1611527, rs11639926	0.967 (0.956–0.977)	1.52^−09^
	PLA2G4B	rs62038328, rs11150438, rs12716929	1.041 (1.027–1.054)	1.86^−09^
	STAG3	rs2967418, rs140439298, rs9304460, rs2161813	1.029 (1.019–1.040)	1.39^−08^
	CTC1	rs1561234, rs114464485, rs12593920	1.018 (1.011–1.024)	9.75^−08^
	HCG11	rs2290556, rs55757218	1.096 (1.059–1.134)	1.59^−07^
	HEATR3	rs112578934, rs4502545	0.963 (0.950–0.977)	2.04^−07^
	LINC00324	rs72615157, rs148654444, rs80044214	1.022 (1.014–1.031)	2.78^−07^
	APEH	rs138791019, rs268461	1.029 (1.018–1.040)	3.11^−07^
	STN1	rs73973191, rs62063070	0.845 (0.791–0.902)	4.02^−07^
	ATP8B4	rs4792590, rs6763931, rs4343916, rs435759, rs9922332, rs116915980, rs3915616	1.042 (1.026–1.059)	4.47^−07^
	IQCG	rs113377106, rs4791747	0.976 (0.966–0.985)	5.68^−07^
	RPA2	rs62063070, rs268461, rs115437298, rs76298543, rs13064576, rs149007767, rs11191865, rs3184504, rs12485444, rs149007767, rs7846314, rs2497306, rs35609972, rs75217875, rs937171, rs2413974, rs116192866, rs76016611, rs13094133, rs74628087, rs113759128, rs142840389, rs113716316, rs12031669, rs144151266, rs141245605, rs17185052	0.983 (0.976–0.990)	1.89^−06^

CI – confidence interval, FI – frailty index, IEAA – intrinsic epigenetic age acceleration, MR – Mendelian randomisation, OR – odds ratio, SNP – single-nucleotide polymorphisms, TL – telomere length

### SMR and HEIDI analysis: validating causal gene expression in ageing biomarkers

Following the two-sample MR analysis, we further validated the robust causal genes for each ageing-related outcome using the SMR and HEIDI test (**Table 2**; Figures S2−5 in the **Online Supplementary Document**). For FI, significant SMR associations were observed for UBA7, LRPPRC, and CRLF3. Similarly, for TL, SMR confirmed significant causal relationships for TGS1, KMT5A, PLA2G4B, STAG3, RPA2, POLI, and IQCG. For Hannum, CD248 demonstrated a significant association. Finally, for IEAA, ATP8B4 exhibited a significant association. Importantly, the directionality of all results was consistent with the initial two-sample MR results. All HEIDI test results were not significant (*P* > 0.05), indicating that these associations are not confounded by linkage disequilibrium. Collectively, the SMR analyses served as an independent validation of our initial MR findings and further reinforced the stability and credibility of the identified causal association. Furthermore, phenome-wide MR profiling linked UBA7 to 19, ATP8B4 to 11, and PLA2G4B to 30 phenotypes (Table S7 in the **Online Supplementary Document**). These phenotypes predominantly represent ageing-related diseases, highlighting potential roles in ageing biology. To further explore translational potential, we queried the DSigDB database for druggable compounds (Table S8 in the **Online Supplementary Document**) [40].

**Table 2:** SMR findings between genes and the four ageing-related traits

	Gene	QTL, source	SMR *P*-value	HEIDI *P*-value
**FI**	UBA7	sQTL, BrainMeta	0.03	0.08
	CRLF3	eQTL, GTEx Whole Blood	1.30^−03^	0.64
		eQTL, Gen	1.97^−06^	0.21
		eQTL, BrainMeta	1.52^−06^	0.35
	LRPPRC	eQTL, GTEx Whole Blood	1.71^−05^	0.65
		eQTL, Gen	8.15^−07^	0.40
		eQTL, BrainMeta	9.42^−07^	0.50
**Hannum**	CD248	eQTL, Gen	5.77^−05^	0.07
**IEAA**	ATP8B4	eQTL, Gen	6.80^−10^	0.42
**TL**	TGS1	eQTL, GTEx Whole Blood	3.90^−11^	0.47
	KMT5A	eQTL, GTEx Whole Blood	2.36^−08^	0.08
		sQTL, BrainMeta	2.92^−09^	0.11
	POLI	pQTL, FENLAND	1.06^−11^	0.24
		mQTL, McRae	1.66^−15^	0.42
	PLA2G4B	eQTL, Gen	1.76^−22^	0.07
	STAG3	eQTL, Gen	8.64^−19^	0.27
		mQTL, BrainMeta	9.07^−08^	0.70
	IQCG	mQTL, McRae	2.62^−04^	0.75
	RPA2	eQTL, BrainMeta	1.00^−09^	0.39
		eQTL, GTEx Whole Blood	3.25^−09^	0.14
		sQTL, BrainMeta	2.08^−09^	0.58

eQTL – expression quantitative trait locus, FI – frailty index, GTEx – genotype-tissue expression, HEIDI – heterogeneity in the dependent instrument, mQTL – methylation quantitative trait locus, MR – Mendelian randomisation, pQTL – protein quantitative trait locus, QTL – quantitative trait loci, SMR – summary-databased MR, sQTL – splicing quantitative trait locus, TL – telomere length

### Cross-cohort validation in independent ancestry groups

To validate the robustness and broad applicability of our findings, we conducted a replication MR analysis on the three target genes – UBA7, PLA2G4B, and ATP8B4 – using GWAS data from additional multiethnic longevity cohorts (**Table 3**). This analysis revealed that UBA7 is significantly associated with longevity, showing higher expression in long-lived populations (*P* = 1.27 × 10^−2^). This result was consistent with our previous finding, suggesting that elevated expression of UBA7 may contribute to extended lifespan.

**Table 3:** Replication findings in the independent cohort

Genes	SNPs	OR (95% CI)	*P*-value
UBA7	rs116375541, rs76298543, rs13086611, rs2236939	1.09 (1.02–1.17)	1.27^−02^
PLA2G4B	rs12593920, rs2290556, rs55757218, rs112578934	1.03 (0.93–1.13)	0.59
ATP8B4	rs12485444, rs149007767, rs7846314, rs2497306, rs35609972, rs75217875, rs937171	1.06 (0.95–1.19)	0.30

CI – confidence interval, OR – odds ratio, SNP – single-nucleotide polymorphisms

### In vivo verification

**Figure 2:**
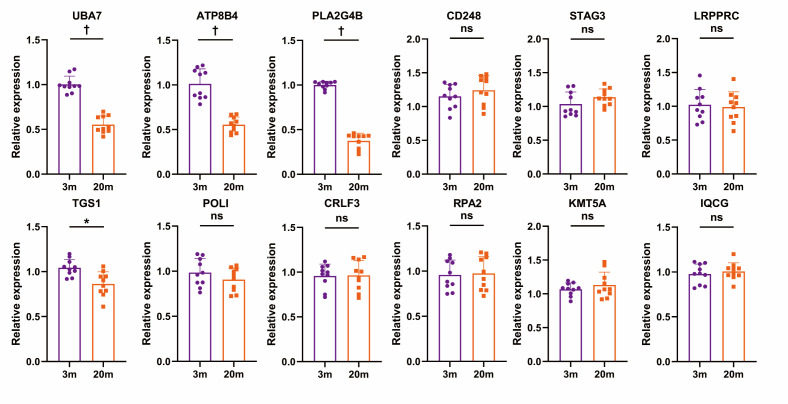
Comparative quantification of mRNA expression for age-related genes in young (3m) *vs.* aged (20m) mice (n = 10). *P ≤ 0.01. †*P* ≤ 0.001. ns – not significant, m – month.

**Figure 3:**
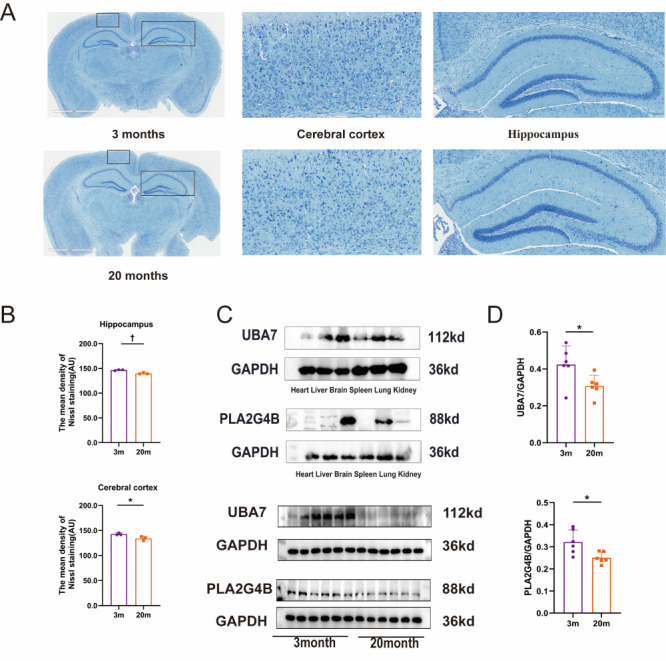
**Panel A.** Representative Nissl-stained images of the cerebral cortex and hippocampus from 3-month-old and 20-month-old mice. **Panel B.** Quantitative analysis of Nissl-stained images comparing the mean density of Nissl-positive signals in the cerebral cortex and hippocampus of 3m and 20m mice (n = 3). **Panel C.** Western blot analysis of UBA7 and PLA2G4B expression across major organs and in the brains of three- and 20-month-old mice (n = 10). **Panel D.** Quantification of Western blot results for UBA7 and PLA2G4B in the brains of three- and 20-month-old mice (n = 10). *P ≤ 0.05. †*P* ≤ 0.01. au – arbitrary units, m – month.

To further validate the age-related expression changes of candidate genes identified through MR analysis, we employed an ageing mouse model using three- and 20-month-old C57BL/6 mice (n/N = 10/group) (**Figure 2**, **Figure 3**). This model is widely used in ageing research to represent young adult and elderly stages in mice [41]. Considering the systemic nature of ageing, we first measured gene expression changes in peripheral blood using qPCR, as peripheral blood transcriptomics can reflect comprehensive physiological alterations across the organism [42]. The results revealed that UBA7, PLA2G4B, and ATP8B4 were significantly down-regulated in aged mice compared to adult mice (*P* < 0.001), consistent with our MR findings. In contrast, POLI, CRLF3, RPA2, and KMT5A exhibited no significant differences between groups, while TGS1 displayed expression patterns inconsistent with MR-based predictions. Additionally, the expression levels of CD248, STAG3, and IQCG were relatively low, and there was no significant difference among the groups, which limited the practicality of further research on these genes. To confirm these findings at the protein level, we performed Western blot analyses focussing on PLA2G4B and UBA7. We did not analyse ATP8B4 due to a lack of antibody. We observed that both proteins were highly expressed in mouse brain tissue, with a significant reduction in expression in aged mice compared to adult controls. These results further corroborated our qPCR findings and bioinformatics predictions.

## DISCUSSION

Ageing is characterised by progressive functional decline and heightened susceptibility to chronic diseases, driven by factors such as chronic inflammation and accumulation of DNA damage. Ageing is also heavily influenced by extrinsic factors such as environmental exposures [43,44], lifestyle [45,46], socioeconomic determinants [47], and access to healthcare. These extrinsic factors interact with intrinsic biological processes, complicating the ageing phenotype and highlighting the need for targeted therapeutic interventions. Despite this complex interplay, effective therapies that directly target the molecular drivers of ageing remain elusive. By combining MR, proteomics, and in vivo validation, we identified causal relationships between several genes and ageing phenotypes, including FI, TL, HannumAge, and IEAA. We found three candidate genes – UBA7, PLA2G4B, and ATP8B4 – that represented promising druggable targets and novel therapeutic avenues for extending lifespan.

We found that UBA7 can delay ageing. According to the literature, it has critical functions in antiviral defence [48], protein homeostasis, and neurodegeneration protection. In contrast to classical ubiquitin-mediated pathways, UBA7 selectively activates ISG15, a ubiquitin-like modifier subsequently transferred by UBE2L6, to coordinate cellular stress responses [48,49]. The UBA7-ISG15 axis is vital for antiviral immunity and homeostasis, as evidenced by UBA7-deficient mice, which exhibit unconjugated ISG15 accumulation, neutrophil-driven inflammation, lung pathology, and mortality [50]. Furthermore, UBA7-mediated ISG15 conjugation can modulate p53 signalling, a process involved in the DNA damage response that may affect genomic integrity and DNA repair, but the mechanisms are not yet fully explained [51]. Environmental changes- such as rhinovirus infection and cigarette-smoke exposure- modulate UBA7 expression in immune cells, indicating that its transcription is environmentally regulated [52,53]. Emerging evidence highlights the diverse roles of UBA7 in ageing and inflammation, underscoring its potential relevance in age-related diseases. Further studies are needed to clarify the underlying molecular mechanisms.

PLA2G4B (*i.e.* cytosolic phospholipase A2 group IVB) is a pivotal regulator of arachidonic acid metabolism with a potential role in modulating the ageing process, similar to UBA7. This gene encodes the enzyme cPLA2-β, which hydrolyses membrane phosphatidylcholines to release arachidonic acid – the precursor for a broad spectrum of pro- and anti-inflammatory lipid mediators essential for immune homeostasis. A recent study has reported significantly decreased PLA2G4B expression in monocytes from elderly populations, coinciding with elevated phosphatidylcholine accumulation [1]. This age-related lipid imbalance may exacerbate the chronic, low-grade inflammation of ageing, now identified as one of the hallmarks of ageing. Furthermore, rare-variant and multi-omics analyses in centenarians indicate that PLA2G4B functional integrity and epigenetic stability are key contributors to exceptional longevity, with loss-of-function mutations in this gene being significantly underrepresented in this long-lived cohort [54]. Consistently, in our gene set enrichment analysis, we found perturbations in pathways related to phospholipid catabolism (GO:0009395) and inflammatory mediator regulation (KEGG hsa04750), aligning with PLA2G4B’s role in lipid mediator turnover and immune regulation. These findings suggest that restoring or enhancing PLA2G4B activity could help rebalance lipid mediator production and immune homeostasis in older adults, potentially mitigating pro-inflammatory drift during ageing. However, given the complexity of arachidonic acid networks and eicosanoid signalling, further in vivo studies are warranted to dissect the underlying mechanisms and to evaluate the safety and efficacy of targeting PLA2G4B as a great strategy.

ATP8B4 is associated with delayed ageing outcomes. As a member of the P4-ATPase family, ATP8B4 plays a crucial role in phospholipid translocation, lipid translocation, and regulation of membrane lipid distribution. Notably, rare damaging variants in ATP8B4 have been linked to an increased risk of Alzheimer disease, which is closely associated with ageing, and early-onset cerebral amyloid angiopathy [55–57]. The cellular and molecular mechanisms of ATP8B4 in ageing remain unclear, and further studies are needed to clarify its precise role and potential as a therapeutic target.

This study has several advantages. First, we employed a comprehensive set of ageing metrics – including FI, epigenetic clocks (HannumAge and IEAA), and TL – as outcomes in our two-sample MR analysis. This approach provides a more nuanced understanding of BA compared to reliance on CA alone. Furthermore, we implemented a multi-level validation strategy by integrating two-sample MR, SMR, and in vivo validation. This methodological rigour enhances the reliability and robustness of our findings. Additionally, replication in independent cohorts reinforces the robustness and generalisability of our MR findings, reducing the risk of false positives and confirming the stability of causal association.

Several limitations should also be noted. First, we primarily included European populations, which may limit the study's relevance to other ethnic groups. Second, the absence of direct age-related measurements necessitated reliance on biomarkers of ageing, which may not comprehensively reflect the multidimensional nature of biological ageing. Consequently, this may lead to incomplete characterisation or systematic biases. Finally, our experimental validation remains preliminary and thus does not fully establish the causality suggested by the MR findings. Despite consistent directional effects across multiple lines of evidence, a limitation is the inability to validate ATP8B4 at the protein level due to the lack of available antibodies, which weakens experimental support and limits interpretability. Future studies involving transgenic models are required to conclusively define the causal roles of the identified genetic variants in ageing.

By integrating genomics with experimental validation, we discovered that UBA7, PLA2G4B, and ATP8B4 can delay ageing. These genes operate within pathways corresponding to key hallmarks of ageing – proteotoxicity, inflammation, and metabolic dysregulation – and represent promising leads for therapeutic targeting. Continued research into these molecular mechanisms could pave the way for interventions to extend health span and mitigate age-related diseases.

## CONCLUSIONS

Our findings highlight UBA7, PLA2G4B, and ATP8B4 as promising targets for interventions to extend health span, illuminating core biological mechanisms of longevity. Future studies using transgenic models are required to conclusively define the causal roles of the identified genetic variants in ageing.

## Additional material


Online Supplementary Document
