# Supplementary material for: Unveiling the genetic biomarkers for ageing: evidence from a large sample genome-wide association study and in vivo validation
Source: J Glob Health. 2025 Sep 26;15:04279. doi: 10.7189/jogh.15.04279 (PMC12467474; doi:10.7189/jogh.15.04279)

Supplement to: Cai Z, Yang Y, Qu P, Fu S, Li X. Unveiling the genetic biomarkers for ageing: evidence from a large sample genome-wide association study and in vivo validation. J Glob Health. 2025;15:04279.

Figure S1. Leave-One-Out Analysis Results

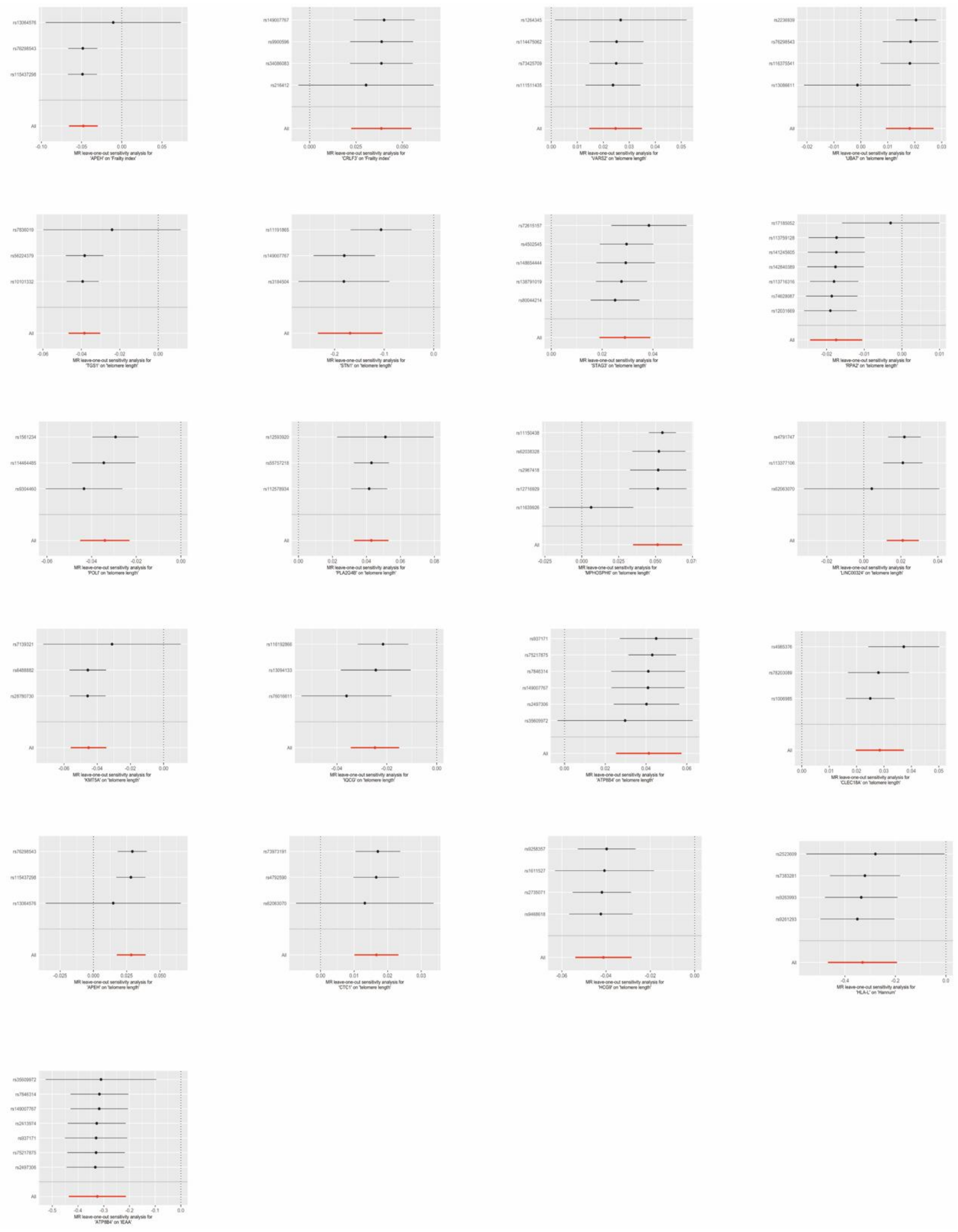

Fig S2 The SMR result of Frailty index

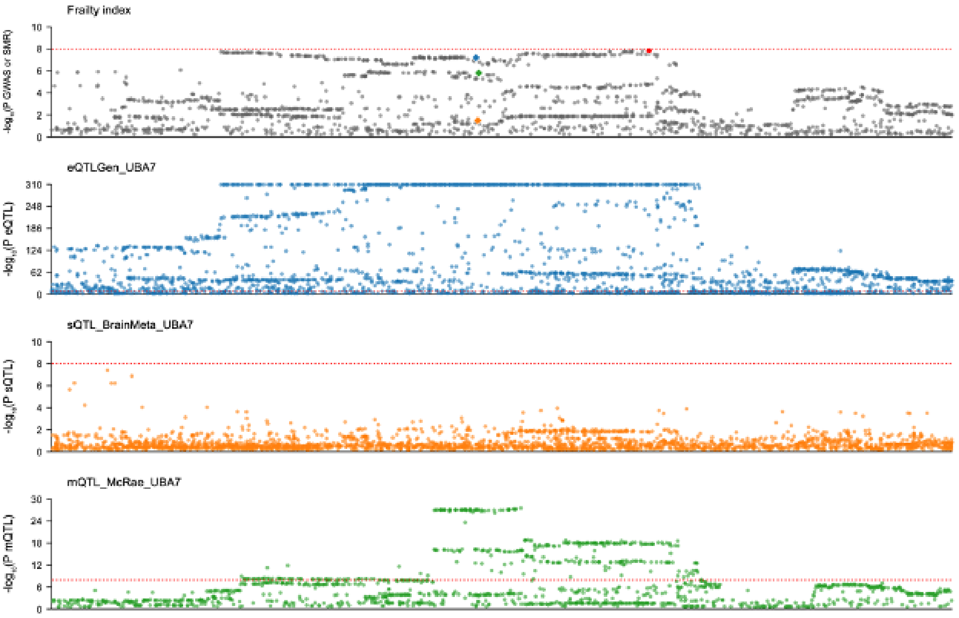

Fig S3 The SMR result of Hannum

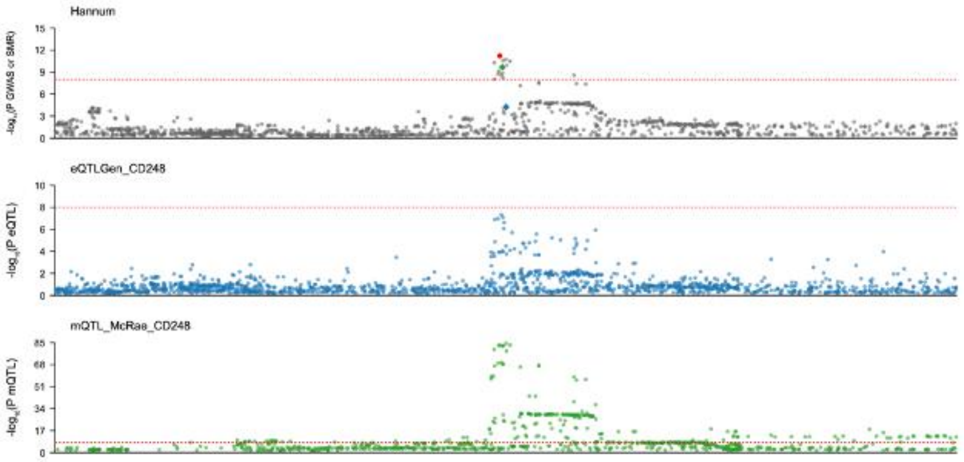

Fig S3 The SMR result of IEAA

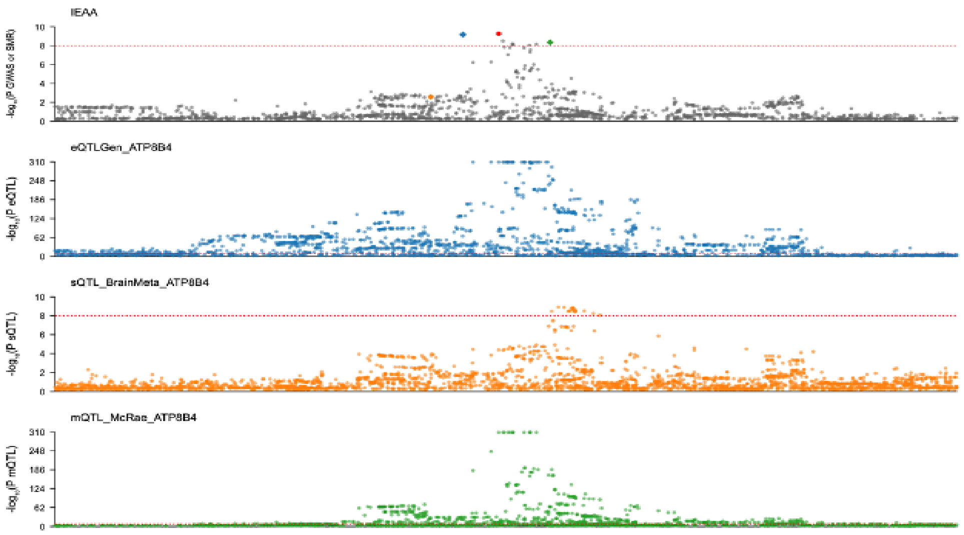

Fig S4 The SMR result of Telomere length

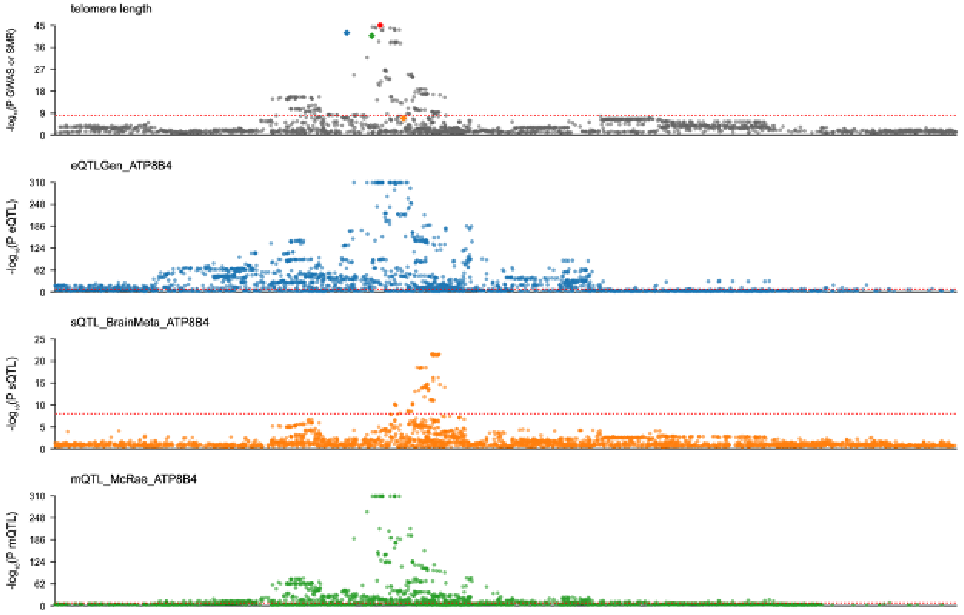

Supplement: Online Supplementary Document [file jogh-15-04279-s001.zip › jogh-15-04279-s001.pdf]
